# Autophagy Induced by Toll-like Receptor Ligands Regulates Antigen Extraction and Presentation by B Cells

**DOI:** 10.3390/cells11233883

**Published:** 2022-12-01

**Authors:** Jonathan Lagos, Sara Sagadiev, Jheimmy Diaz, Juan Pablo Bozo, Fanny Guzman, Caroline Stefani, Silvana Zanlungo, Mridu Acharya, Maria Isabel Yuseff

**Affiliations:** 1 Laboratory of Immune Cell Biology, Department of Cellular and Molecular Biology, Pontificia Universidad Católica de Chile, Santiago 8331150, Chile; 2 Center of Immunology and Immunotherapies, Seattle Children’s Research Institute, Seattle, WA 98101, USA; 3 Núcleo Biotecnología Curauma, Pontificia Universidad Católica de Valparaíso, Valparaíso 2373223, Chile; 4 Benaroya Research Institute at Virginia Mason, Seattle, WA 98101, USA; 5 Department of Gastroenterology, School of Medicine Pontificia Universidad Católica de Chile, Santiago 8331150, Chile; 6 Department of Pediatrics, University of Washington, Seattle, WA 98195, USA

**Keywords:** B cell, antigen extraction, TLR, CpG, autophagy, αV integrin, LPS

## Abstract

The engagement of B cells with surface-tethered antigens triggers the formation of an immune synapse (IS), where the local secretion of lysosomes can facilitate antigen uptake. Lysosomes intersect with other intracellular processes, such as Toll-like Receptor (TLR) signaling and autophagy coordinating immune responses. However, the crosstalk between these processes and antigen presentation remains unclear. Here, we show that TLR stimulation induces autophagy in B cells and decreases their capacity to extract and present immobilized antigens. We reveal that TLR stimulation restricts lysosome repositioning to the IS by triggering autophagy-dependent degradation of GEF-H1, a Rho GTP exchange factor required for stable lysosome recruitment at the synaptic membrane. GEF-H1 degradation is not observed in B cells that lack αV integrins and are deficient in TLR-induced autophagy. Accordingly, these cells show efficient antigen extraction in the presence of TLR stimulation, confirming the role of TLR-induced autophagy in limiting antigen extraction. Overall, our results suggest that resources associated with autophagy regulate TLR and BCR-dependent functions, which can finetune antigen uptake by B cells. This work helps to understand the mechanisms by which B cells are activated by surface-tethered antigens in contexts of subjacent inflammation before antigen recognition, such as sepsis.

## 1. Introduction

In vivo, B cells efficiently recognize antigens tethered at the surface of antigen-presenting cells (APC), such as macrophages or follicular dendritic cells [1,2], forming a domain known as an immune synapse (IS). IS formation is initiated by the engagement of antigens to the B cell receptor (BCR), triggering downstream signaling, actin cytoskeleton rearrangements [3,4] and the recruitment of key organelles, such as centrosomes and lysosomes towards the synaptic membrane [5]. Physical properties, such as the stiffness of the membrane where antigens are presented [6] can determine their mode of extraction. Antigens coupled to softer surfaces are internalized by myosin II-dependent pulling forces [7], whereas antigens associated with substrates with high stiffness are acquired by proteolytic extraction by means of lysosome secretion [5,8]. The secretion of lysosomes at the synaptic membrane relies on the assembly and recruitment of the exocyst complex, which is regulated by the Rho GTP exchange factor, GEF-H1 [9].

In addition to mechanical cues, other extracellular signals from the microenvironment can also affect the antigen extraction capacity of B cells. For instance, Galectin-8, which is expressed in the lymphoid microenvironment, was shown to promote antigen extraction through a proteolytic mechanism [10]. Moreover, stimulation via Toll-like receptors (TLR), which recognize patterns associated with microbial pathogens, also impacts B cell functions in multiple ways. Dual BCR and TLR engagement triggered by viral antigens or autoantigens can promote B cell proliferation, class switching, and entry to germinal centers [11,12]. However, the exposure of B cells to TLR9 ligands, such as CpG, before antigen recognition decreases their antigen extraction and antigen-presenting capacity [13]. In B cells, TLR activation is regulated by integrin αVβ3, a vitronectin receptor that regulates responses to growth factors and chemokines [14]. During recognition of TLR ligands, αV integrins trigger the conjugation of endosomes with the autophagy protein LC3 through a Src-Syk pathway [15,16], promoting LC3 lipidation at the membrane of endosomes and fusion with lysosomes to end TLR signaling. Consequently, the absence of this integrin decreases the conjugation of TLR-containing endosomes with LC3, impairing their fusion with lysosomes. Additionally, the activation of TLRs can also trigger changes in the cortical actin cytoskeleton of B cells. Stimulation with LPS induces severing of the actin cytoskeleton, increasing the mobility and interactions of cell surface BCRs, thereby promoting signaling responses [17]. Thus, TLR stimulation has an impact on the diverse pathways that shape B cell activation.

Autophagy is an evolutionarily conserved process used by cells to recycle damaged or unwanted intracellular cargo, and is activated during cellular stress or starvation [18]. During canonical autophagy, cytoplasmic content is recruited to double membrane vesicles conjugated with LC3, which enables their fusion with lysosomes and the posterior degradation of their contents. Additionally, autophagy machinery and LC3 are involved in other signaling and trafficking events, in which single membrane vesicles are conjugated with LC3 to enable the fusion of their cargo with lysosomes by a process referred to as non-canonical autophagy [19,20]. The role of autophagy in B cells is complex, being involved in different stages, such as the differentiation and self-renewal of B1 cells, maintenance of plasma cells during antibody production and maintenance of memory B cells [21,22,23,24]. Recent evidence has shown that autophagy proteins, such as Atg5, are required by B cells to extract immobilized antigens [25]. Additionally, BCR stimulation triggers non-canonical autophagy, and components of the autophagy machinery are recruited to endosomes containing internalized antigens [26]. In other immune cells, such as macrophages, TLR stimulation induces non-canonical autophagy, which regulates the phagocytosis of extracellular antigens [27]. In B cells, however, the role of autophagy induced by TLR ligands during antigen recognition is less understood.

In this work, we address how autophagy, induced by TLR ligands, regulates the ability of B cells to extract and present immobilized antigens. Our results show that TLR-stimulated B cells display higher levels of autophagy and lower antigen extraction and presentation capacities. TLR stimulation leads to the degradation of GEF-H1, which impairs the stable recruitment of lysosomes at the immune synapse, thereby decreasing antigen extraction by B cells. We provide evidence that the degradation of GEF-H1 is an autophagy-dependent process that relies on αV integrin. B cells that do not express αV integrin conserve their antigen extraction capacity in the presence of CpG. Altogether, these results highlight how TLR ligands tune the antigen extraction and presentation capacity of B cells by controlling autophagy and might represent a mechanism to prevent the proliferation of autoreactive lymphocytes.

## 2. Materials and Methods

### 2.1. Cells and Mice

The mouse IgG+ B-Lymphoma cell line IIA1.6 [28,29] and the LMR7.5 T cell hybridoma that recognize I-Ad-Lack156–173 complexes [30,31,32] were obtained from Dr. Lennon-Dumenil (Institute Curie, Paris, France). The cells were cultured in enriched RPMI (RPMI 1640 with Glutamax supplemented with 10% heat-inactivated FBS, 0.1% 2-mercaptoethanol, 100 µg/mL penicillin, 100 µg/mL streptomycin, and 1 mM sodium pyruvate). The human diffuse large IgM+ B cell lymphoma (HBL-1) cell line was provided by Dr. Richard James (Seattle Children’s Research Institute, Seattle, WA, USA). αV-CD19 mice were previously described [33] and maintained on a mixed C57bl/6/129Ola background. Littermates with a single CD19-cre allele and Itgav-flox alleles were used as controls. Splenocytes were harvested in phosphate-buffered saline (PBS)/0.5% bovine serum albumin (BSA)/2 mM EDTA and depleted of red blood cells (RBC lysis buffer, Sigma-Aldrich, Burlington, MA, USA).

All mice were housed under specific pathogen–free conditions at Seattle Children’s Research Institute. All animal experiments were approved following institutional review by the Animal Care and Use Committees at Seattle Children’s Hospital Research Institute. Experiments were performed under local and national guidelines for animal care.

### 2.2. Antibodies and Reagents

Antibodies used for immunofluorescence are rat anti-LAMP1 (BD Pharmigen, Franklin Lakes, NJ, USA, #553792, 1:200), rabbit anti-CEP55 (Abcam, #ab170414, 1:500), rabbit anti-OVA (Sigma-Aldrich; #C6534, 1:500), rat anti–α-tubulin (Abcam; #ab6160, 1:500), rabbit anti-LC3 (Novus biological, NB100-2220, 1:200) and rabbit anti–GEF-H1 (Abcam; #ab155785, 1:500). The following secondary antibodies were used: Alexa Fluor 488-, Alexa fluor 568 Cy3 and Alexa Fluor 647—conjugated F(ab’)2 donkey anti-rat and Cy3-conjugated F(ab’)2 donkey anti-rabbit (Jackson ImmunoResearch, West Grove, PA, USA 1:500); and Alexa Fluor 488-conjugated goat anti-rabbit (Molecular Probes, Invitrogen, Eugene, OR, USA 1:200). F-actin was stained using Alexa Fluor 546- or Alexa Fluor 647- conjugated phalloidin (Life Technologies, Carlsbad, CA, USA, #A22287, 1:200). For western blot, the following antibodies were used: anti–γ-tubulin (Abcam, Boston, MA, USA; #Ab11317, 1:1000); mouse anti-actin (MP Biomedicals, Santa Ana, CA, USA; clone C4; #691001); rabbit anti-LC3 (Novus biological, Englewood, CO, USA; NB100-2220, 1:1000); rabbit anti–GEF-H1 (Abcam; #ab155785, 1:1000); and rabbit anti–LAMP-1 (Cell Signaling, Danvers, MA, USA; #C54H11, 1:1000). As a secondary antibody, HRP-conjugated donkey anti-mouse, anti-rat or anti-rabbit (Jackson ImmunoResearch; 1:5000) was used.

The Lack antigen was produced and purified from bacteria through histidine tag purification with Ni-NTA agarose beads (Invitrogen), and the Lack peptide (aa 156–173) was synthesized by PolyPeptide Group. For autophagy induction, Torin 1 300 nM was used (Tocris, Bristol, UK). To stimulate TLRs we used LPS 1 µg/mL or CpG 1 µM.

### 2.3. Preparation of Antigen-Coated Beads and Antigen-Coated Coverslip

Antigen-coated beads were prepared as previously described [5]. Briefly, ∼2 × 10^7^ 3-μm latex NH2-beads (Polyscience, Eppelheim, Germany) were activated with 8% glutaraldehyde for 4 h at room temperature. Beads were washed with phosphate-buffered saline (PBS) and incubated overnight at 4 °C with different ligands: 100 μg/mL of F(ab’)2 goat anti-mouse immunoglobulin G (anti-IgG), as BCR-Ligand+ referred as BCR-Ligand+ for IIA1.6 B cells, or 100 μg/mL of F(ab’)2 goat anti-mouse IgM, referred to as BCR-Ligand− for IIA1.6 or BCR-Ligand+ for primary B cells (Jackson Immunoresearch). As BCR-Ligand+ for HBL1 B cells we use 100 μg/mL of F(ab’)2 goat anti-human IgM (Jackson Immunoresearch). For antigen extraction assays, beads were coated with BCR-Ligand+ or BCR-ligands− plus OVA 100 μg/mL. For antigen presentation assays, beads were coated with BCR+ or BCR− ligands plus 100 μg/mL Lack protein. Antigen coverslips used to analyze the synaptic interface were coated with BCR-Ligand+ overnight at 4 °C in PBS at 100 μg/mL.

### 2.4. Immobilized Surface Antigens

For analysis of the synaptic plane, IIA1.6 B cells were plated onto BCR-ligand+ coated glass coverslips, for different time points and incubated at 37 °C, as previously described [34]. For activation with BCR ligand-coated beads, cells were plated on poly-L-lysine-coated slides and stimulated with the beads at a 1:1 ratio (cells to beads) for the indicated times at 37 °C, and fixed in 4% paraformaldehyde for 10 min at room temperature. Fixed cells were incubated for 60 min with primary antibodies and 60 min with secondary antibodies in PBS-BSA-saponin (1×/0.2%/0.05%). Coverslips were mounted onto slides using Fluoromount G (Electron Microscopy Sciences, Hatfield, PA, USA).

### 2.5. Antigen Extraction

For antigen extraction assays, IIA 1.6 or primary B cells incubated in a 1:1 ratio with BCR ligand+-OVA-coated beads were plated on poly-Lys covered slides at 37 °C, fixed and stained for OVA. The amount of OVA remaining on the beads was calculated by establishing a fixed area around beads in contact with cells and measuring fluorescence on three-dimensional (3D) projections obtained from the sum of each plane (details in Image Analysis section). The percentage of antigen extracted was estimated by the percentage of fluorescence intensity lost by the beads after 30 or 60 min.

### 2.6. Antigen Presentation

Ag presentation assays were performed as previously described [5]. Briefly, IIA 1.6 (I-Ad) B cells were incubated with either Lack-BCR-Ligand+ or BCR-Ligand− coated beads or different concentrations of Lack peptide (Lack156−173) for 2 or 4 h. Then, cells were washed with PBS, fixed in ice-cold PBS/0.01% glutaraldehyde for 1 min, and quenched with PBS/100 mM glycine. B cells were then incubated with Lack-specific LMR 7.5 T cells in a 1:1 ratio for 4 h. Supernatants were collected, and interleukin-2 cytokine production was measured using BD optiEA Mouse IL-2 ELISA set following the manufacturer’s instructions (BD Biosciences, Franklin Lakes, NJ, USA).

### 2.7. Activation of B Cells on Antigen-Coated Plates and Western Blot Densitometric Analysis

CpG or LPS pre-treated HBL1 B cells were placed on six-well culture plates previously coated overnight with 100 μg/mL of F(ab′)2 goat anti-human IgM (Jackson Immunoresearch) diluted in cold PBS and then processed to be analyzed by western blot. Band intensity was determined by ImageJ. Normalization of protein expression was performed by calculating the density of the target protein, multiplied by the ratio between the density of the loading control from the control sample (time 0 or untreated) and the density of loading control from the lane of interest. The fold change was calculated by dividing the normalized expression from each lane by the normalized expression of the control sample.

### 2.8. CRISPR Targeting

CRISPR strategy and validation targeting ATG5 and ITGAV were previously described [35]. Briefly, CRISPR crRNAs were identified using the Broad Institute single guide RNA design tool and synthesized (Integrated DNA technologies, Coralville, IA, USA). crRNAs and trans-activating crRNA (tracrRNA: 1,072,532) hybrids were mixed with Cas9 nuclease (IDT, 1,081,058) at a ratio of 1.2:1 and transfected into cells by Neon electroporation (Thermo Fisher Scientific, Bothell, WA, USA, MPK1025). The transfected cell mixture was singlecell sorted, and total genomic DNA was extracted from the single-cell clones using Quick-DNA Microprep Kit (ZymoResearch, Irvine, CA, USA; D3021). Knockout efficiency was assessed by amplifying guide target genomic regions using PrimeSTAR GXL DNA Polymerase (Takara Bio, San Jose, CA, USA; R050A). PCR products were purified using the GeneJet PCR Purification Kit (Thermo Fisher Scientific, K0701) and analyzed by Sanger Sequencing and Inference of CRISPR. Edits (Synthego, Redwood City, CA, USA). Flow cytometry or western blot was used to validate knockdown on a protein level. (Guides ATG5: CCUUAGAUGGACAGUGCAGA; ITGAV: AGCCGAAGUAACUUCCCUCG; tracrRNA binding sequence: GUUUUAGAGCUAUGCU).

### 2.9. Microscopy

Epifluorescence microscopy images were acquired using a 63×/1.4 NA oil-immersion objective on an Eclipse Ti microscope (Nikon instrument, Melville, NY, USA) equipped with an iXon Ultra EMMCCD camera (Oxford Instruments, Abingdon, UK) and operated by NIS-Elements Advanced Research imaging software. Confocal images were obtained using a 100×/1.45 NA oil-immersion objective on an inverted Nikon-Ti2 equipped with a CMOS ORCA-Flash 4.0 V3 camera and operated by NIS-Elements Advanced Research imaging software in the Advanced Microscopy Facility UMA UC. Analysis in primary B cells was performed using a Nikon Ti Eclipse Ultraview Spinning-Disk confocal (CSU-X1) with a 100×/1.4 NA oil-immersion objective and an Orca-ER camera, operated by Volocity software. HBL1 cells were imaged on a 63× oil objectives (aperture 1.4) on Leica TCS SP5 confocal microscope (Leica, Wetzlar, Germany).

### 2.10. Image Analysis

Images were analyzed in Image J Fiji distribution 2.9 [36], macro scripts were generated in-house, and they are available at https://github.com/Jonathan-Lagos/scriptsforfiji2022 (accessed on 6 November 2022).

#### 2.10.1. Lysosome and GEF-H1 Recruitment

Accumulation of lysosomes or GEF-H1 at the IS was quantified by measuring the LAMP1, LAMP2, or GEF-H1 fluorescence intensity in a concentric circular area surrounding the bead (3 µm) [9]. In the case of GEF-H1, levels in this area were normalized by fluorescence at the bead at time 0 min. For spreading analysis, we manually delimited the border of the cell using the phalloidin label as a template, then a ⅓ of the cell area ellipse was generated in the center by the macro script. Next, the recruitment of structures to the center of the immune synapse was calculated by dividing the fluorescence of LAMP1 or LC3 in the center of the cell normalized by its area by the fluorescence in the total area normalized by its area, subtracting 1. More positive values indicate more enrichment of the fluorescence label in the central area.

#### 2.10.2. Polarity Analysis

LAMP-1 polarity indexes were calculated as described previously [5]. Briefly, using macro scripts in Fiji, we manually selected the cell and the bead area. Then, from these areas, the cell and bead centers were extracted. For the lysosomes, we measured the center of mass of LAMP-1 staining. The distance between the center of mass of Lamp1 (CL) and the center of the cell (CC) was projected onto the distance of the center of the bead and the center of the cell (CB) multiplying by the cosine of the angle between both distances described. The polarity index was calculated by dividing the projection of CL by CB. The index ranged from −1 (anti-polarized) to 1 (fully polarized).

#### 2.10.3. Distribution of Lysosomes within Z Sections

The distribution of lysosomes within Z sections was previously described [37]. Values of lysosomes were calculated by measuring their mean fluorescence intensity (MFI) in the cell at each z-slice (0.2 µm) from the immune synapse (defined by the contact area on antigen coated dishes) to the top of the cell.

Data obtained from the images were then curated and filtered using Python to posterior statistical analysis.

### 2.11. Flow Cytometry

IIA1.6 pretreated with CpG (24 h), LPS (24 h) or Torin 1 (2 h) were stained with FITC anti-mouse I-A^d^ Antibody (Biolegend, San Diego, CA, USA, 1:100) to label surface MHC-II. Samples were acquired using FACS Canto II flow cytometry (BD Biosciences, Franklin Lakes, NJ, USA) and analyzed using the FlowJo software (Tree Star Inc, Ashland, OR, USA).

### 2.12. Statistical Analysis

Data were collected from 3 independent experiments, except when specified otherwise, and reported as mean ±SEM. Statistical analysis was performed using Student’s t test or one- or two-way ANOVA and post hoc analysis for multiple comparisons (specified in figure legends) using Prism (GraphPad Software, San Diego, CA, USA). The *p*-values were computed using different tests, as indicated in figure legends; * 0.01 < *p* < 0.05, ** 0.001 < *p* < 0.01; *** *p* < 0.001; ns, no significant.

## 3. Results

### 3.1. TLR Stimulation Decreases the Capacity of B Cells to Extract and Present Immobilized Antigens by Decreasing Lysosome Recruitment to the Immune Synapse

**Figure 1:**
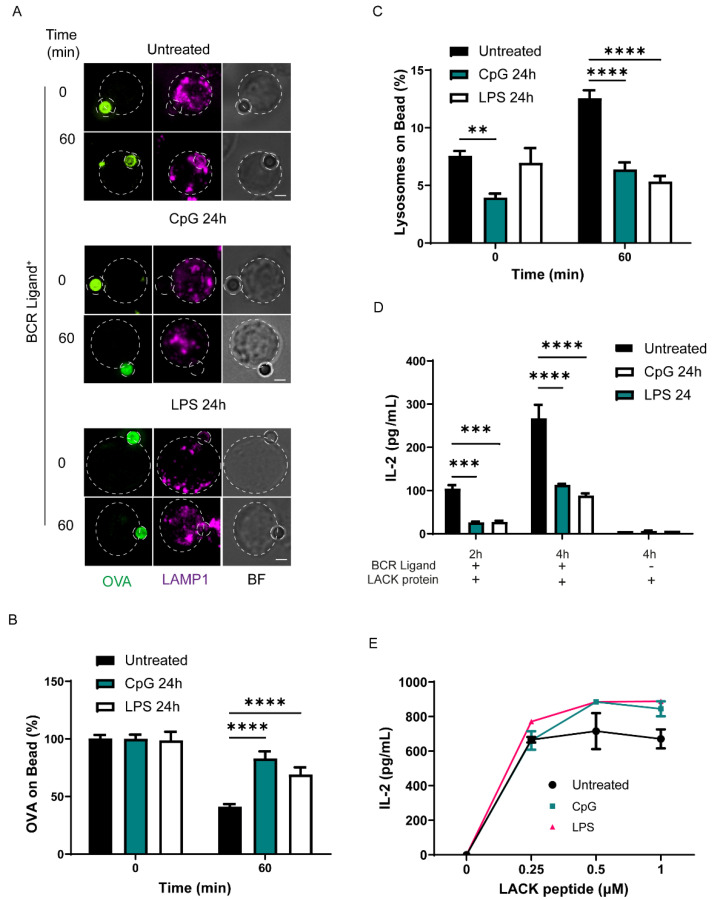
Treatment with CpG and LPS decrease antigen extraction and presentation by B cells. (**A**) Representative images of control, CpG and LPS pre-treated B cells incubated with beads coated with OVA and a BCR ligand+ (anti-IgG) in resting (0 min) and activated (60 min) conditions. Cells were fixed and stained for OVA (green) and LAMP-1 (magenta). BF: Bright Field. Images are shown as Z-projections of a stack. Scale bar = 3 µm. N = 3 (**B**) Quantification of OVA fluorescence remaining on the bead, normalized by initial fluorescence. *** *p* < 0.001, **** *p* < 0.0001. N = 3. (>90 cells). Two-way ANOVA with Sidak’s multiple comparison test. (**C**) Quantification of lysosome accumulation at the IS. ** *p* < 0.01, **** *p* < 0.0001. N = 3. (>90 cells). Two-way ANOVA with Sidak’s multiple comparison test. (**D**) Antigen presentation assay of control, CpG or LPS pre-treated B cells. Levels of IL-2 secretion by T cells were quantified by ELISA, *** *p* < 0.001 **** *p* < 0.0001. N = 3. Two-way ANOVA with Sidak’s multiple comparison test. (**E**) Representative graph of peptide controls for cells used in antigen presentation assays.

TLR ligands upregulate autophagy and stimulate antigen-presenting cells by increasing surface levels of MHC-II and costimulatory molecules [38]. However, previous work has shown that B cells decreased their ability to extract and present immobilized antigens after treatment with the TLR9 ligand, CpG [13]. We sought to evaluate the underlying mechanisms behind the effect of TLR stimulation on antigen extraction and presentation and focused on lysosomes, considering that polarized secretion of lysosomes can facilitate the extraction of antigens at IS of B cells [5]. For this purpose, we pretreated IIA1.6 B cells with 1 µM of CpG or 1 µg/mL of LPS for 24 h, under the conditions previously described, to modify the intracellular trafficking of lysosomes in B cells and other immune cells [16,27,39], and evaluated the antigen extraction capacity of these cells. To this end, B cells treated or not treated with TLR ligands were incubated with ovalbumin (OVA) and BCR ligand+ (anti-IgG for these cells) coated beads, which mimics the formation of an immune synapse (IS), for different periods of time. Antigen extraction was evaluated by measuring the fluorescence signal of ovalbumin (OVA) remaining on the bead after 60 min of activation in comparison with initial conditions (0 min). Our results show that after 60 min, beads interacting with B cells pretreated with CpG or LPS presented higher levels of remaining OVA, in comparison to control cells (Figure 1A,B). In agreement with previous studies [13], these results suggest that B cells diminish their capacity to extract antigens when previously exposed to TLR ligands. Importantly, antigen extraction was not observed in B cells incubated with beads conjugated to OVA plus anti-IgM as a negative BCR ligand (Figure S1A,B), showing that this process requires BCR activation. Considering that lysosome secretion can facilitate antigen extraction from latex beads [5,8], we evaluated the local recruitment of lysosomes near the bead, which represents the IS [9]. To this end, B cells were pretreated for 24 h with TLR ligands and stained for Lamp1+ lysosomes after incubation with activating BCR ligand+ beads for 60 min. The recruitment of lysosomes was evaluated by measuring the fluorescence of LAMP1 accumulated in the bead, as previously described [40]. Our results show that B cells treated with TLR ligands recruit fewer lysosomes to the IS upon BCR stimulation (Figure 1C). This result was confirmed by calculating lysosome polarity indexes, as a measurement of their proximity to the IS [34], which also revealed that fewer lysosomes were recruited to the IS of B cells treated with TLR-ligands (Figure S2A) and explains why these cells display decreased antigen extraction. Our results suggest that this was not due to an overall decrease in the amount of lysosomes, given that total levels of LAMP1 measured by immunoblot of lysates obtained from B cells pretreated with CpG or LPS revealed no significant differences (Figure S2B,C). Importantly, lysosome polarization was not observed in B cells incubated with beads coupled to negative BCR ligand (Figure S1A,C,D). To further evaluate the functional impact of TLR ligands in B cells, we performed an antigen presentation assay in the presence or absence of TLR ligands as a readout to determine the antigen extraction and processing capacity of B cells. We incubated B cells pretreated with TLR ligands (CpG 1 uM or LPS 1 μg/mL) for 24 h and next incubated these cells for 2 h or 4 h with BCR+ beads containing the Lack antigen from Leishmania major. The ability of B cells to present MHC-II peptide complexes derived from bead-associated Lack to a specific T hybridoma was then measured by monitoring IL-2 secretion by activated T cell lymphocytes. As expected, the presentation of bead associated Lack antigen to T cells decreased when B cells were treated with TLR ligands (Figure 1D), which correlated to their deficiency in antigen extraction capacity. Noticeably, TLR ligands improved the ability of B cells to present peptides (Figure 1E), suggesting that cell surface levels of MHC class II increased upon treatment with TLR ligands as we confirmed by Flow Cytometry (Figure S2D). Altogether, our data show that the extraction and presentation of immobilized antigens by B cells significantly declines in the presence of TLR ligands.

### 3.2. TLR Stimulation Regulates the Distribution of Lysosomes at the Immune Synapse

**Figure 2:**
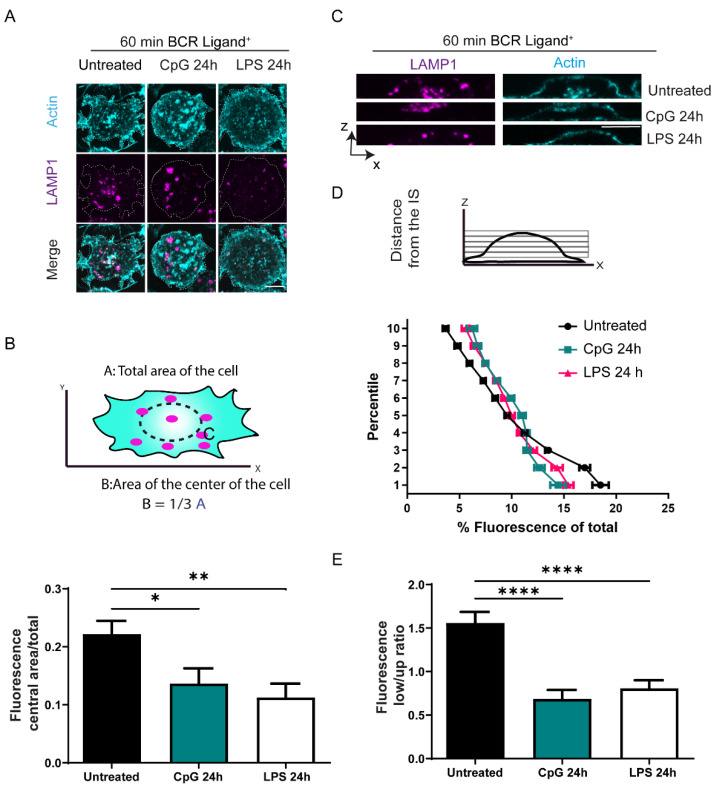
Lysosome distribution at the IS of B cells pre-treated with CpG or LPS. (**A**) Representative confocal images of control, LPS, and CpG pre-treated B cells activated on antigen-coated coverslips for 60 min stained for Lysosomes, LAMP1 (magenta) and F-actin, phalloidin (Cyan) (**B**) Scheme depicting central and total areas defined in the B cell synapse. Graphs represent the ratio between central and total area. * *p* < 0.05, ** *p* < 0.01, N = 3 (>35 cells). One-way ANOVA with Dunnet’s multiple comparison test. (**C**) representative X/Z confocal images from control, CpG, and LPS pre-treated B cells activated on antigen-coated coverslips for 60 min, stained for Lysosomes, LAMP1 (magenta) and F-actin, phalloidin (Cyan). (**D**) Quantification of the LAMP1 fluorescence along the Z dimension from the coverslip to the upper cell limit of control, CpG and LPS pre-treated cells. N = 3 (>38 cells). (**E**) Graphs represent the ratio between the bottom half of the cell and the upper half of the cell from cells shown in C. **** *p* < 0.0001, N = 3 (>38 cells). One-way ANOVA with Dunnet’s multiple comparison test.

Our results show that B cells treated with TLR ligands do not efficiently recruit lysosomes to the IS. We investigated this effect further and analyzed the distribution of lysosomes at the synaptic interface under TLR stimulation. For this purpose, B cells pre-treated or not with TLR ligands were plated onto glass slides coated with a BCR ligand+ and incubated for 60 min. Images taken by confocal microscopy of the synaptic plane of control B cells showed that lysosomes are recruited at a central area. As reported in previous work, this area most likely corresponds to the central supramolecular activation complex (cSMAC) where BCRs also gather [41]. In contrast, lysosomes from B cells pre-treated with CpG or LPS presented a more peripheral localization at the synaptic interface (Figure 2A,B). Lysosome distribution in the whole cell, was evaluated by taking XZ images of B cells activated on antigen-coated coverslips and labeled for Lamp1. The fluorescence signal for Lamp1 was measured across the Z dimension, as shown in Figure 2C. Measurements corresponding to lysosomes in each Z plane revealed a progressive enrichment of lysosomes at the synaptic interface of control cells. In contrast, we found that B cells treated with CpG or LPS presented fewer lysosomes in planes next to s and were concentrated further away from IS (Figure 2D). Indeed, the ratio of fluorescence distribution of lysosomes between the lower (Fluorescence low) and upper (Fluorescence up) halves of the cell decreased in CpG or LPS-treated B in comparison to control cells (Figure 1E). Thus, TLR stimulation decreases lysosome recruitment to the IS, which is correlated with their poor antigen extraction and presentation capacity.

### 3.3. Autophagy Is Increased in B Cells Stimulated with TLR Ligands

**Figure 3:**
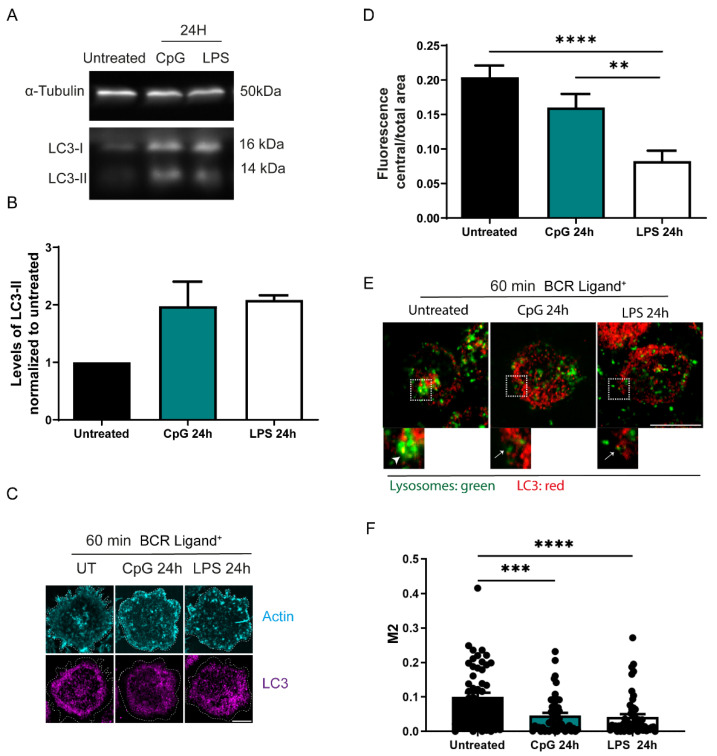
TLR ligands induce autophagy in B cells and affect lysosomal distribution. (**A**) Immunoblot showing levels of LC3-II in control, CpG and LPS pre-treated cells. (**B**) Quantification of the ratio of LC3-II and α-Tubulin from A. N = 3. (**C**) Representative confocal images of control, LPS, and CpG pre-treated B cells activated on antigen-coated coverslips for 60 min and stained for LC3 (magenta) and F-actin, phalloidin (Cyan). (**D**) Quantification of the ratio between the central and total area of the B cell synapse (>38 cells). ** *p* < 0.01, **** *p* < 0.0001, N = 3 (>35 cells). One-way ANOVA with Dunnet’s multiple comparison test. (**E**) Confocal images on the synaptic plane showing control, CpG and LPS pre-treated B cells activated at on an antigen-coated coverslip for 60 min and stained for LAMP1 (Green) and LC3 (Red). Insets highlight LAMP1 and LC3 localization in the center or periphery of the immune synapse. Arrowhead shows colocalization of LC3 and LAMP1 near the center of the IS, meanwhile arrows show LC3 and LAMP1 interacting in the periphery of the cell. Scalebar 10 µm. (**F**) Manders’ coefficient of fraction of lysosomes overlapping with LC3. One-way ANOVA with Dunnet’s multiple comparison test (>50 cells); *** *p* < 0.001, **** *p* < 0.0001.

Lysosome distribution is modified by autophagic flux [42], and therefore, we investigated whether TLR engagement affects lysosomal distribution via autophagy. To investigate this, we first assessed whether TLR stimulation promotes the induction of LC3-II, the marker for activation of autophagy and monitored LC3 distribution in B cells. Indeed, we observed increased levels of LC3-II in B cells exposed to TLR ligands, CpG and LPS (Figure 3A,B). We confirmed this result by immunofluorescence, where we stained LC3 and evaluated the formation of LC3 puncta, which indicates their effective lipidation in vesicle membranes. We found more puncta in cells treated with CpG or LPS in comparison to control B cells, regardless of activating conditions with BCR ligands (Figure S3A), suggesting that TLR ligands increase autophagy in B cells. Considering that lysosomes in TLR-stimulated cells displayed a peripheral distribution, we next evaluated whether LC3 distribution was also modified in B cells treated with CpG or LPS, 24 h prior to BCR activation. Indeed, in B cells treated with LPS or CpG, LC3 was also localized more at the periphery in comparison to control cells (Figure 3C,D), where colocalization of lysosomes with LC3 at the synaptic plane also decreased, suggesting that there could be an increased consumption of LC3 close to lysosomes (Figure 3E,F, arrows) in comparison to untreated cells that display higher colocalization of LAMP1 with LC3 at the center of the synapse (arrowheads). Additionally, B cells treated with CpG or LPS presented lower levels of LC3 in the planes nearest to the IS (Figure S3B). Overall, these results show that TLR engagement increases autophagy modifying the distribution and recruitment of LC3 to the immune synapse.

### 3.4. Induction of Autophagy Decreases the Capacity of B Cells to Extract and Present Immobilized Antigen

**Figure 4:**
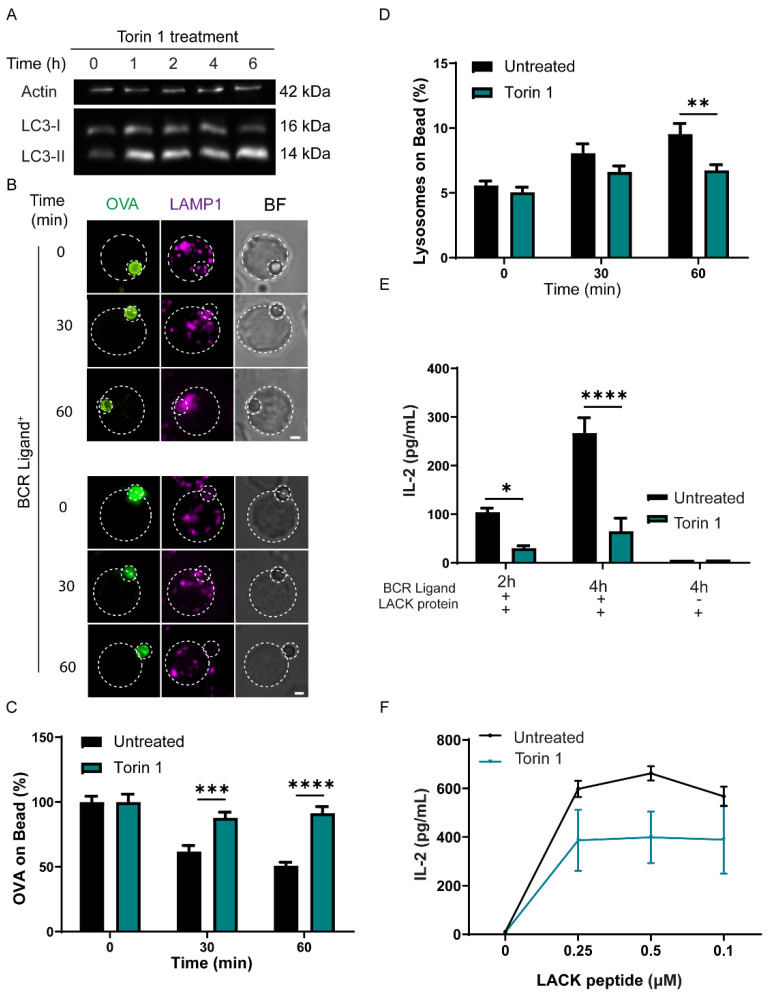
Induction of autophagy, independently of TLR stimulation, decreases antigen extraction and presentation by B cells. (**A**) immunoblot showing levels of LC3-II in B cells treated with Torin 1 at different time points; a representative blot of 3 independent experiments is shown. (**B**) Representative images of control and Torin 1-treated B cells incubated with beads coated with OVA and BCR ligand+ (anti-IgG) for different periods of time. Cells were fixed and stained for OVA (green) and LAMP-1 (magenta). Images are shown as Z-projections of a stack. Scale bar = 3 µm. N = 3. (**C**) Quantification of OVA fluorescence remaining on the bead, normalized by initial fluorescence. *** *p* < 0.001, **** *p* < 0.0001. N = 3. (>55 cells). Two-way ANOVA with Sidak’s multiple comparison test. (**D**) Quantification of lysosome accumulation at the IS. ** *p* < 0.01. N = 3. (>55 cells). Two-way ANOVA with Sidak’s multiple comparison test. (**E**) Antigen presentation assay of control or Torin 1-treated B cells. Levels of IL-2 secretion by T cells were quantified by ELISA * *p* < 0.05, **** *p* < 0.0001. N = 3. (**F**) Representative graph of peptide controls for cells used in antigen presentation assays.

To directly determine the effect of autophagy on antigen extraction and presentation, we increased autophagy, independently of TLR ligands, and evaluated these functions in B cells. For this purpose, we incubated B cells with Torin 1, an antagonist of the mTOR pathway which increases autophagy (Figure 4A). B cells incubated with 300 nM of Torin 1 increased the levels of LC3-II after 1 h of treatment, an indicator of autophagy induction. Importantly, this treatment did not induce cell death in B cells, as determined by staining for annexin V/Propidium iodide, which was similar to control cells (Figure S4A,B). Next, antigen extraction was evaluated by incubating B cells treated or not with Torin 1, with OVA-BCR ligand+ beads for different periods of time, and the amount of OVA remaining on beads was measured, as described above. As shown in Figure 4B,C, cells treated with Torin 1 presented less antigen extraction in comparison to control cells. Torin 1-treated B cells also displayed a lower percentage of lysosomes at the bead interface under these conditions (Figure 4C), suggesting that lysosome recruitment to the IS diminished upon induction of autophagy. Accordingly, we found defective lysosome polarization to the IS, as indicated by polarity indexes, which were lower compared to control cells after 60 min of activation (Figure S4A). Using non-stimulating beads, we did not observe changes in lysosome dynamics (Figure S5B–D). Furthermore, Torin 1-treated B cells also displayed lower antigen presentation capacity, when incubated with BCR+ beads containing Lack antigen, similarly to assays with TLR ligands (Figure 4F). Interestingly, treatments with Torin 1 also decreased the capacity of B cells to present processed peptides to T cells without affecting the level of MHC II molecules at the cell surface (Figure S5E), suggesting that Torin 1-treated B cells display loaded MHC-II molecules, preventing interaction with the soluble Lack peptides. To determine whether the induction of autophagy was a general mechanism that regulates the antigen presentation capacity of cells, we evaluated these functions under starvation, which also triggers autophagy. We found that starvation also decreased antigen extraction (Figure S5A,B) and antigen presentation by B cells (Figure S5C,D), without affecting their capacity to present processed peptides. In summary, autophagy induction decreases the ability of B cells to extract and present immobilized antigens efficiently.

### 3.5. Antigen Extraction in B Cells Is Regulated by TLR-Induced Autophagy via Integrin αV

**Figure 5:**
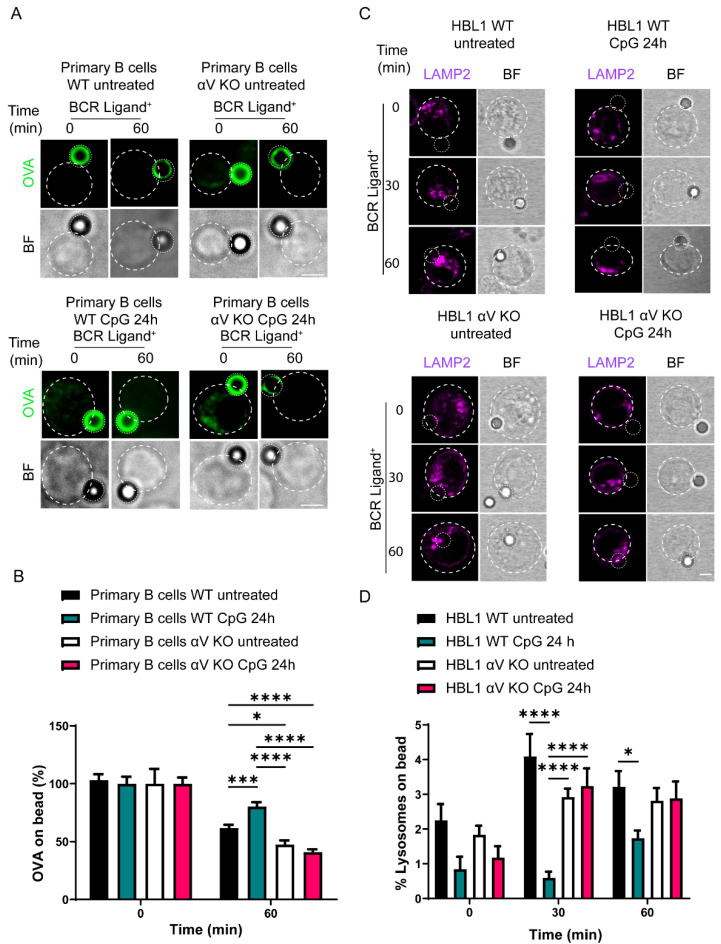
Antigen extraction is regulated via TLR-induced autophagy and is mediated by αV integrin. (**A**) Representative confocal images of control and CpG pre-treated primary B cells incubated with beads coated with OVA and BCR ligand+ (anti-IgM) for resting (0 min) and (60 min). One plane of representative images is shown. (**B**) Quantification of OVA fluorescence remaining on the bead, normalized by initial fluorescence. * *p* < 0.05, *** *p* < 0.001, **** *p* < 0.0001. N = 3. (>90 cells). Two-way ANOVA with Sidak’s multiple comparison test. N = 3. (>78 cells) (**C**) confocal images of control and CpG pre-treated HBL1 B cells incubated with beads coated with OVA and BCR ligand+ in resting (0 min) and activated (60 min) conditions and stained for LAMP2 to label lysosomes). One plane of representative images is shown. (**D**) Quantification of lysosome accumulation at the IS. * *p* < 0.05, **** *p* < 0.0001. N = 3. (>90 cells). Two-way ANOVA with Sidak’s multiple comparison test. N = 3. (>30 cells).

We next investigated whether impaired antigen extraction in B cells exposed to TLR ligands depended on their capacity to induce autophagy. For this purpose, we used primary B cells from an αV KO mouse which exhibited impaired autophagy induction upon TLR engagement [16]. Primary B cells isolated from control and KO mice were evaluated in terms of their capacity to extract OVA from beads in the presence or absence of TLR stimulation. Our results showed that primary B cells from control mice extracted less antigen upon treatment with CpG, similar to our findings in the B cell line. Interestingly, αV KO B cells treated with CpG did not decrease their antigen extraction capacity; instead, antigen extraction was rather increased compared to WT cells (Figure 5A,B). Given that studying changes in lysosomal distribution in primary B cells is challenging due to their small size, to investigate lysosome polarization, we used a human B cell line HBL1 CRISPR-Cas9 knocked down for integrin αV [35]. As expected, WT B cells pre-treated with CpG recruited fewer lysosomes to the IS, compared to untreated WT cells. In contrast, CpG pre-treated αV KO cells conserved their ability to polarize lysosomes to the IS, similarly to untreated cells, (Figure 5C,D). Together, our results showed that αV KO B cells, which are unable to induce autophagy upon TLR stimulation did not show a defect in their capacity to recruit lysosomes to the IS when they were pre-treated with CpG, conserving their capacity to extract antigens in the presence of this ligand. Together, these results suggest that autophagy induced by TLR regulates antigen extraction by diminishing the recruitment of lysosomes to the immune synapse.

### 3.6. TLR-Induced Autophagy Controls Levels of GEF-H1, a Regulator of Lysosome Trafficking in B Cells

**Figure 6:**
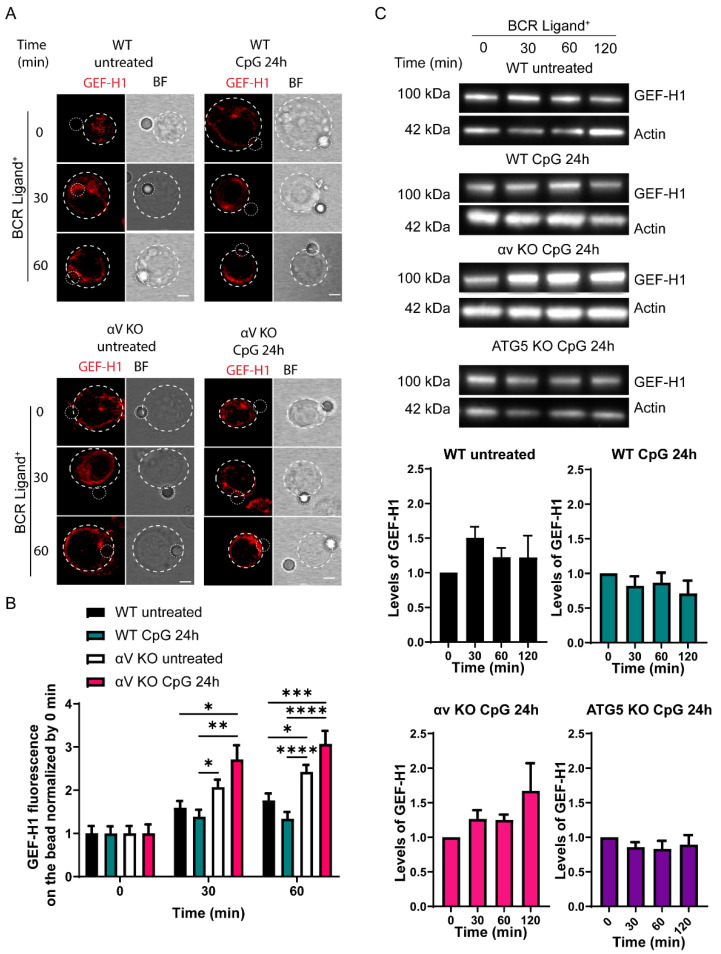
Autophagy induced by TLR stimulation decreases the levels of GEF-H1 at the IS. (**A**) Representative confocal images of control and CpG pre-treated B cells stained for GEF-H1 (red). (**B**) Fold change of GEF-H1 at the area of the bead, normalized by the bead fluorescence at time 0. * *p* < 0.05, ** *p* < 0.01, *** *p* < 0.001, **** *p* < 0.0001. N = 3. (>100 cells). Two-way ANOVA with Sidak’s multiple comparison test. (**C**) Immunoblot showing levels of GEF-H1 in control and CpG pre-treated cells, activated on antigen-coated plates at different time points. Bottom: quantification of GEF-H1 levels normalized by time 0. N = 3.

We next focused on how autophagy regulates lysosome polarization to the IS. For this reason, we investigated the role of GEF-H1, which has been described as being involved in the tethering and fusion of lysosomes at the immune synapse. GEF-H1 is a microtubule-associated Rho GTP exchange factor that regulates RhoA activation in response to RalA GTPase, which in turn regulates the localization and assembly of exocyst components [43]. In B cells, GEF-H1 controls both the assembly and recruitment of the exocyst complex to the synaptic membrane, thereby enabling lysosome tethering and secretion to facilitate antigen extraction and presentation [9]. Interestingly, the levels of GEF-H1 were shown to be controlled by autophagy in fibroblasts [44]. Therefore, we asked whether levels of GEF-H1 were also regulated similarly in B cells. To this end, we evaluated whether the recruitment of GEF-H1 to the IS was affected upon activation with TLR ligands by imaging analysis. We observed less GEF-H1 in the synaptic area of CpG-treated control B cells. Surprisingly, αV KO B cells displayed higher levels of GEF-H1 at the IS compared to control cells, and recruitment was more pronounced in αV KO B cells pretreated with CpG (Figure 6A,B). Next, we evaluated whether the reduced recruitment of GEF-H1 to the IS was due to lower levels of GEF-H1 caused by increased levels of autophagy in cells stimulated with CpG. For this purpose, we measured the levels of GEF-H1 in B cells with or without autophagy induced by CpG under BCR engagement and resting conditions. Control and αV KO HBL1 cells were pretreated for 24 h with CpG, then seeded on BCR-ligand+ plates for different periods of time, and levels of GEF-H1 were measured by Western Blot. As shown in Figure 6C, untreated control cells showed an increase in the levels of GEF-H1 at 30 min of BCR activation and then a decrease and stabilization of their levels at later time points. CpG-treated wild-type cells have lower levels of GEF-H1 at all time points of BCR activation compared to untreated cells. In contrast, αV KO B cells which have a defect in TLR-induced autophagy showed higher levels of GEF-H1 during BCR activation, suggesting that the degradation of this protein is regulated by autophagy induced by TLR ligands. To confirm the role of autophagy in GEF-H1 degradation, we performed a similar experiment in HBL1 cells where ATG5, an essential autophagy factor, was knocked out using CRISP-Cas9. As expected, the levels of GEF-H1 did not change significatively upon BCR stimulation, confirming that the autophagy machinery regulates GEF-H1 degradation (Figure 6C). Overall, these results show that levels of GEF-H1 are reduced in CpG-treated B cells by induction of TLR-associated autophagy. Consequently, lower levels of GEF-H1 are recruited to the IS, thus explaining why lysosomes do not efficiently accumulate at the synaptic membrane upon treatment with TLR ligands.

## 4. Discussion

Our results show that autophagy induced by stimulation with TLR ligands has a direct impact on the extraction and presentation of immobilized antigens by B cells. TLR ligands are present in different pathogens and are essential to activating antigen-presenting cells in the context of infection. In this work, we showed that antigen extraction and the presentation of B cells in the context of TLR stimulation is regulated by autophagy, and a delay of autophagy induced by TLR ligands such as in αV KO cells could increase antigen extraction.

The generation of germinal centers depends on the ability of B cells to extract and present antigens. TLR stimulation has been described to improve germinal center responses to produce high-affinity class-switched antibodies [45]. This increase in germinal center responses contrasts with the work by Akayya et al., which showed decreased antigen extraction and presentation in B cells treated with CpG. These differences could be due to the difference in the timing of antigen recognition and TLR engagement. Molecules that engage both BCR and TLR will promote synergic signaling due to common pathways between both receptors, thereby improving antigen recognition [46]. Meanwhile, if TLR stimulation occurs before BCR engagement, the autophagy machinery will activate mechanisms to deviate lysosomes to terminate TLR signaling, at the expense of lysosomes that would be recruited to the IS. Our work supports this last scenario and could help to understand how antigen recognition and B cell activation are affected in a subjacent inflammatory setting such as sepsis or autoimmune disease. We propose here that by modulating autophagy, TLR ligands can tune the ability of B cells to extract and present immobilized antigens.

Autophagy is a complex mechanism, playing different roles in immune cells. Previous studies have shown the importance of autophagy machinery, Atg5, in the capacity of B cells to extract and present immobilized antigens [25], but the cellular mechanisms underlying this effect remained unknown. Additionally, autophagy is a source of intracellular antigens and autoantigens [47,48,49] and thus the concomitant activation of canonical autophagy with antigen extraction could be detrimental to the cell in sorting antigens from intracellular versus extracellular sources, leading to the presentation of autoantigens. Tuning such pathways could impact B cell differentiation in response to autoantigens from pathogenic antigens. Additionally, antigen extraction and autophagy may compete for resources because both pathways use common components, such as lysosomes and autophagy proteins. For instance, other cell types, such as retinal epithelial cells, use LC3-associated phagocytosis (LAP) to capture photoreceptor outer segments (POS). However, upon starvation, both the uptake and endocytosis of POS are diminished [50], showing that LAP and autophagy are incompatible during the internalization of extracellular components.

Autophagy plays an important role in the presentation of intracellular antigens [51], viral antigens [48,49], and autoantigens [52] which rely on the fusion of autophagosomes with MHC-II compartments [53]. In B cells, the induction of autophagy promotes the presentation of intracellular, cytosolic, and nuclear proteins [54]. Interestingly, we observed that B cells treated with Torin 1 present less soluble Lack peptides to T cells, despite having similar surface levels of MHC-II compared to untreated cells. It is tempting to speculate that Torin 1-treated B cells contain MHCII molecules loaded with other peptides, originating from intracellular sources [55]. Dendritic cells from animal models with a deletion in Atg5 are impaired in antigen presentation [56], but since this protein has several functions which are not directly associated with autophagy, such as vesicle secretion and microtubule remodeling [25,35,57], more studies are required to understand the role of autophagy proteins such as ATG5 in antigen processing and presentation. Overall, several lines of evidence indicate that the induction of autophagy supports the presentation of cytosolic antigens. Here, we bring forward an additional mechanism, by showing that autophagy promotes the degradation of GEF-H1, which prevents lysosome polarization to the immune synapse of B cells, diminishing the capture of new extracellular antigens. Another important observation is that TLR signaling promotes a peripheral distribution of lysosomes at the IS of B cells. In dendritic cells, the activation of TLR4 by LPS promotes lysosome tubulation and localization of MHC-II to the plasma membrane, supporting antigen presentation [58]. In B cells, we did not observe lysosome tubulation; however, we found more LAMP1+ vesicles in the periphery of cells. Previous work showed that LAMP1+ vesicles in the periphery have a rapid degradative capacity and are competent in antigen presentation because they contain MHC-II and cathepsin-S [59]. The classical pathway where antigens are internalized into multivesicular bodies to encounter MHC-II molecules [29] requires more time to process and present the antigen in comparison to these peripheral LAMP1+ vesicles. The implication of two separate pathways is not known, but differences in the repertoire of the peptides presented in each one of these compartments cannot be excluded [60]. We found differences in the quantity of lysosomes at the IS in autophagy-induced cells, but differences in the quality of these lysosomes remain unaddressed.

Our work also revealed the role of the integrin αV in the localization of lysosomes in B cells. Connections between integrins and microtubules regulate the transport of vesicles to specific sites, such as focal adhesions, fibrillar adhesions and immune synapses [61]. Interestingly, focal adhesions (FA) are regulated by autophagy [62,63] and recent studies showed that microtubule depolymerization stabilizes FA by promoting GEF-H1-dependent activation of RhoA and myosin IIA [64]. We speculate that αV integrin at the immune synapse could regulate of GEF-H1 by interacting with microtubules. Connection between microtubules and integrins could help to target the autophagy machinery to FAs, promoting the degradation of FA and GEF-H1, which would also impair the tethering and secretion of lysosomes at these zones. Results presented in this work suggest that the integrin αV acts as a break during antigen recognition at the IS. The role of this integrin seems to be conflicting with LFA-1 (αLβ2), which stabilizes B cell-APC interactions and promotes actin cytoskeleton remodeling to enhance antigen recognition [65,66]. It would be interesting to elucidate the interplay between both integrins during immune synapse formation to see whether they are activated sequentially to regulate different phases of IS formation and function in B cells. Altogether, this work contributes to the understanding of the diverse mechanisms that tune B cell responses to antigens and how they impact activation in contexts such as infectious diseases and autoimmunity.

## 5. Conclusions

B cells stimulated with TLR ligands induce autophagy and diminish their antigen extraction and presentation capacity. B cells that do not express integrin αV and are deficient in TLR-induced autophagy conserve their ability to extract immobilized antigens after TLR stimulation. The effect of TLR ligands on antigen extraction results from autophagy-dependent degradation of GEF-H1, required for the recruitment of lysosomes to the immune synapse. Consequently, B cells stimulated with TLR ligands recruit fewer lysosomes to the immune synapse and extract immobilized antigens less efficiently.

## Data Availability

The original contributions presented in the study are included in the article and Supplementary Material; further inquiries can be directed to the corresponding author/s.

## Supplementary Materials

The following supporting information can be downloaded at: https://www.mdpi.com/article/10.3390/cells11233883/s1. Figure S1: B cells incubated with BCR Ligand beads fail to polarize lysosomes efficiently to the IS; Figure S2: Lysosomal polarization, levels of LAMP1 and MHC-II molecules of CpG and LPS pre-treated B cells; Figure S3: TLR stimulation induces autophagy in B cells; Figure S4: Torin 1 does not affect B cell viability; Figure S5: Torin 1-treated B cells fail to polarize lysosomes to the IS; Figure S6: Induction of autophagy by starvation is sufficient to decrease antigen extraction and presentation in B cells.
