# Supplementary material for: Autophagy Induced by Toll-like Receptor Ligands Regulates Antigen Extraction and Presentation by B Cells
Source: Cells. 2022 Dec 1;11(23):3883. doi: 10.3390/cells11233883 (PMC9741325; doi:10.3390/cells11233883)
Supplement: Supplementary file 1 [file cells-11-03883-s001.zip › cells-2029995-supplementary.pdf]

Supplementary materials for:

Autophagy induced by Toll Like Receptor Ligands Regulates Antigen extraction and presentation by B cells

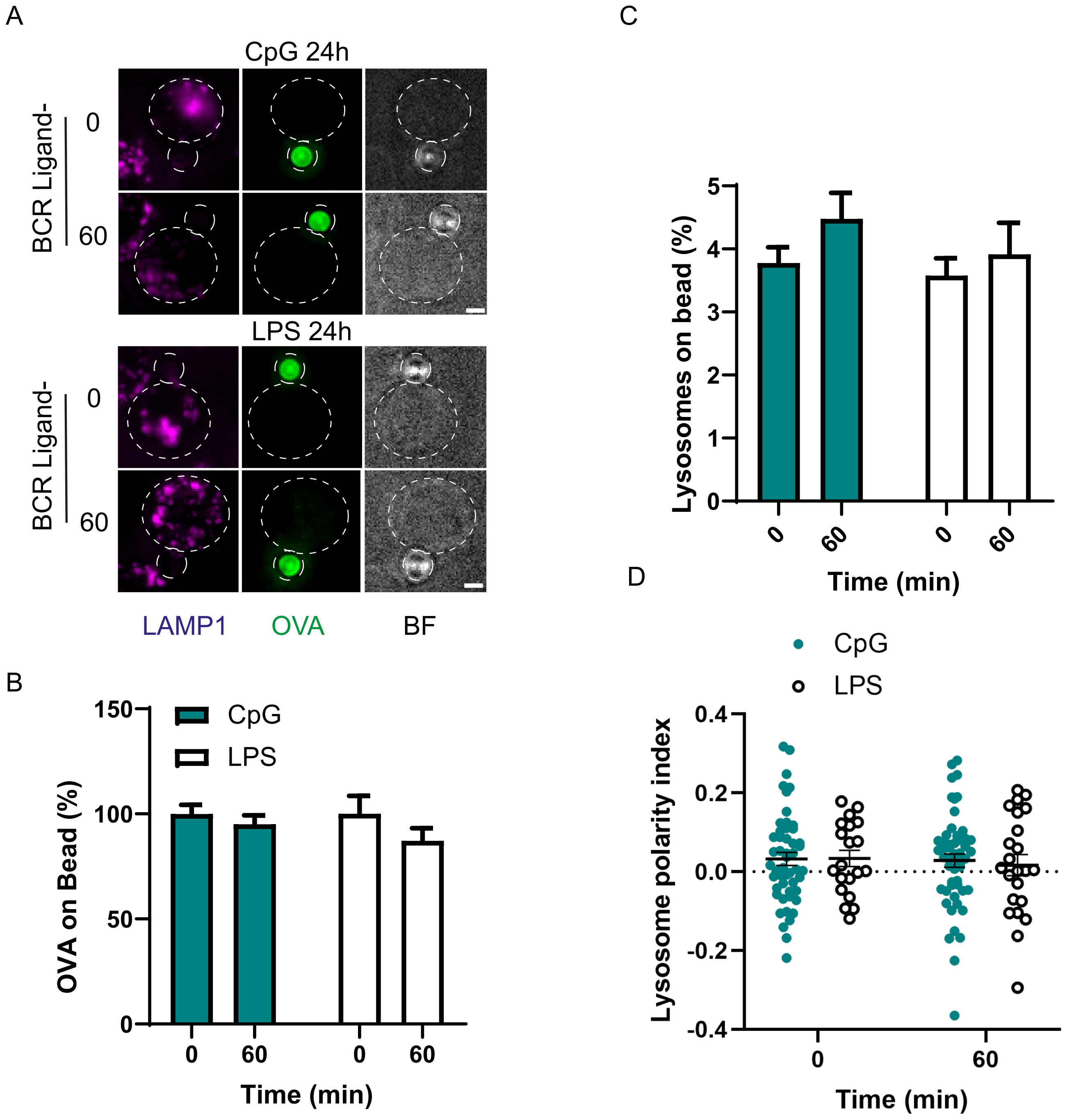

Figure S1. B cells incubated with BCR Ligand- beads fail to polarize lysosomes efficiently to the IS. (A) Representative images of CpG and LPS pre-treated B cells incubated with beads coated with OVA and a BCR ligand- (anti-IgM) in resting (0 min) and activated (60 min) conditions. Cells were fixed and stained for OVA (green) and LAMP-1 (magenta). Images are shown as Z-projections of a stack. Scale bar = 3 μm. N=2 (B) Quantification of OVA fluorescence remaining on the bead, normalized by initial fluorescence. (C) Quantification of lysosome accumulation at the IS. (D) Lysosome polarity indexes of cells of Figure S2 B.

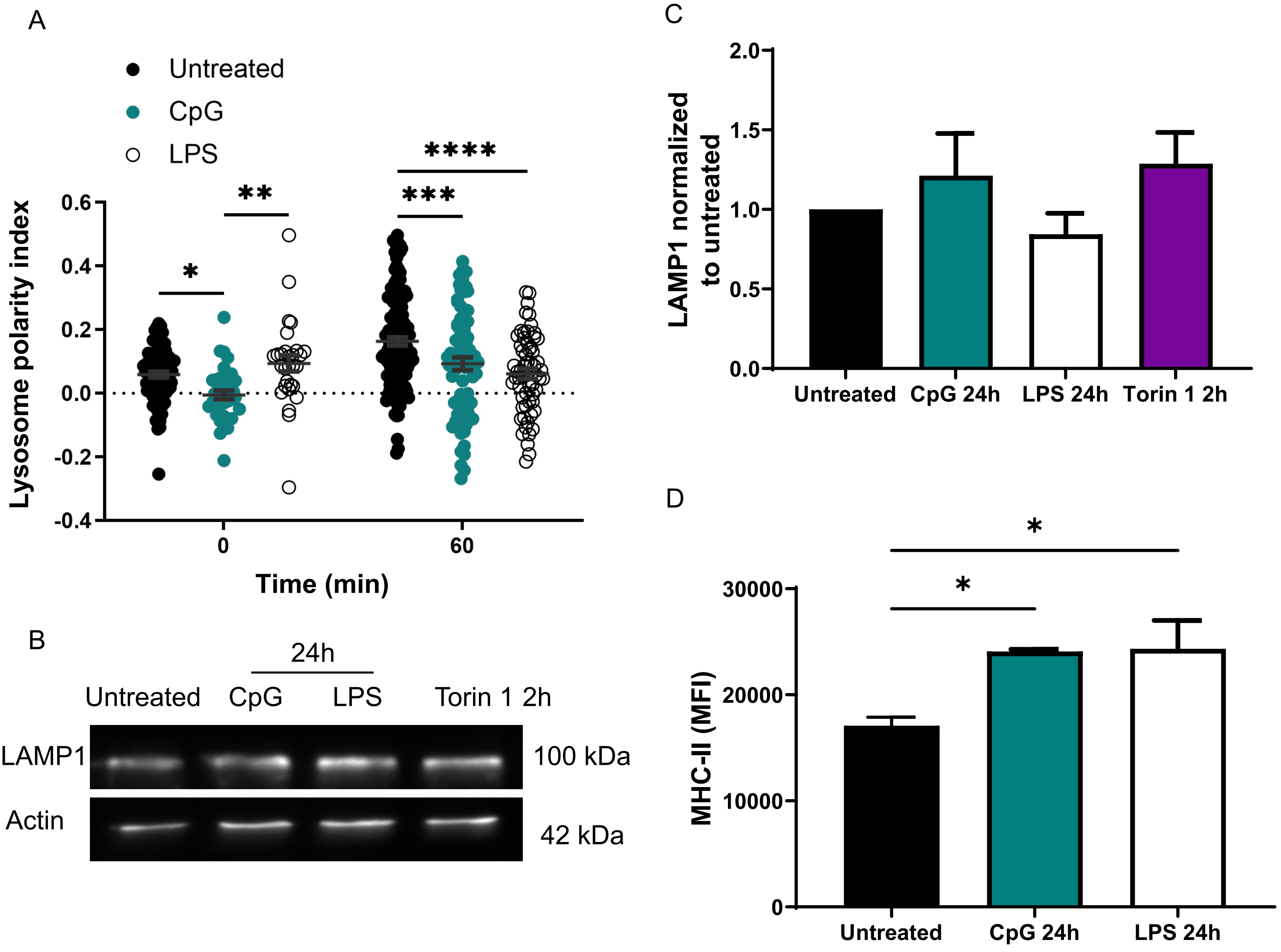

Figure S2. Lysosomal polarization, levels of LAMP1 and MHC-II molecules of CpG and LPS pre-treated B cells. (A) Lysosome polarity indexes of cells from Figure 1A. *p<0.05, **p<0.01, ***p<0.001, ****p<0.0001. N=3. (>90 cells). Two-way ANOVA with Sidak's multiple comparison test. (B) Immunoblot showing levels of LAMP1 in control, CpG, LPS, and Torin 1 treated B cell. (C) Quantification of the ratio of LAMP1 and Actin from B. (N=2). (D) Mean fluorescence intensity of MHC-II of untreated CpG and LPS pre-treated B cells. One way-ANOVA (N=3).

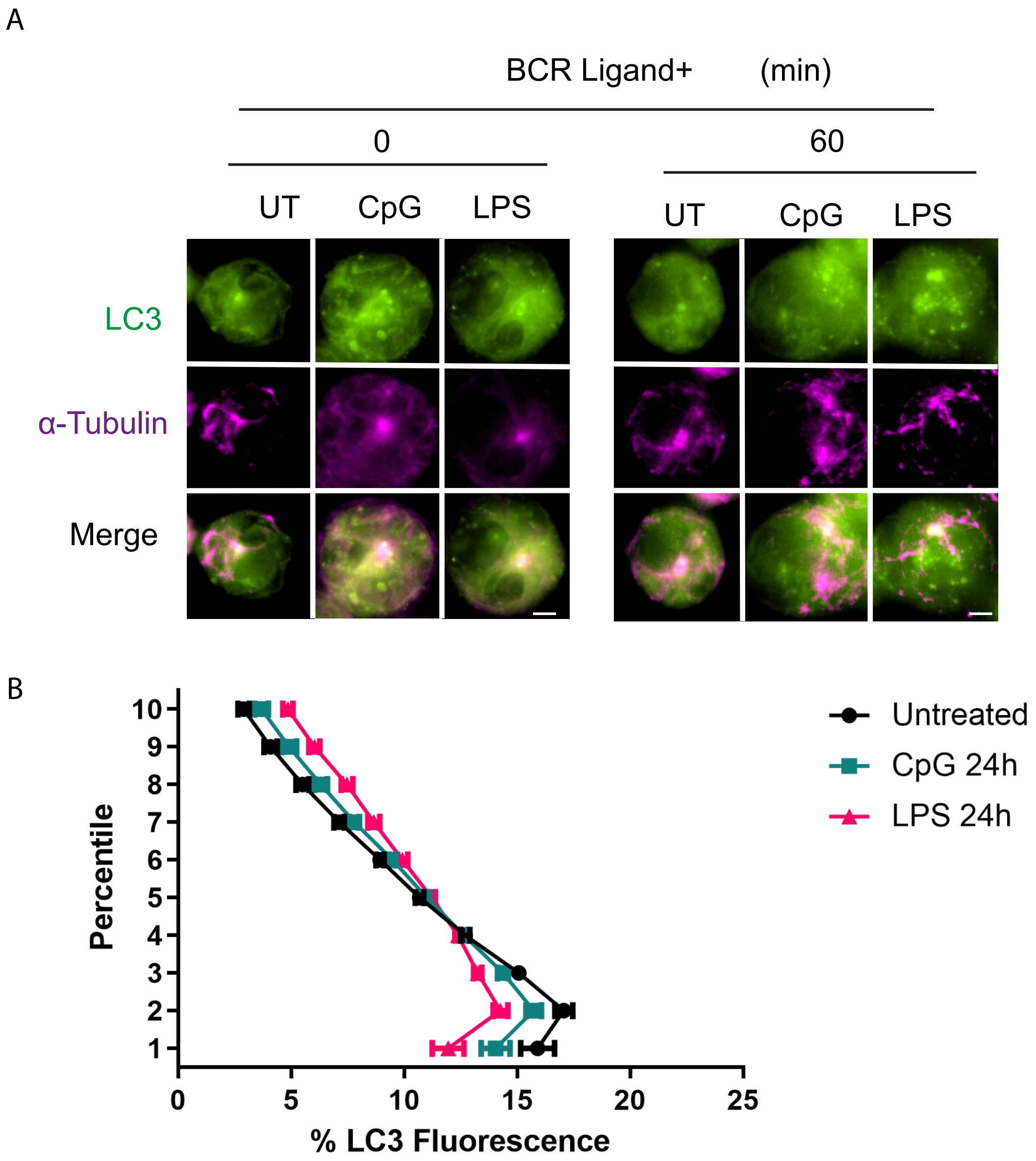

Figure S3. TLR stimulation induces autophagy in B cells. (A) Confocal images of control, or CpG or LPS pre-treated B cells in resting (0 min) or activated condition (60 m) showing LC3 (green) and α-Tubulin (magenta) staining. One plane of representative images is shown. (B) Quantification of LAMP1 fluorescence along the Z dimension from the coverslip to the upper cell limit of control, CpG, and LPS pre-treated cells. N=3 (>38 cells).

A

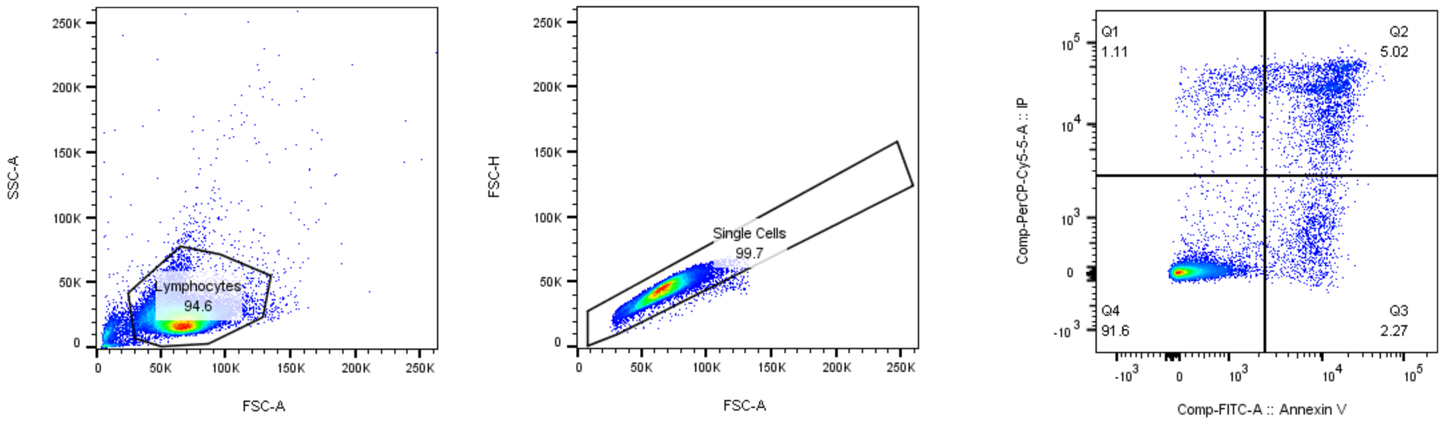

B

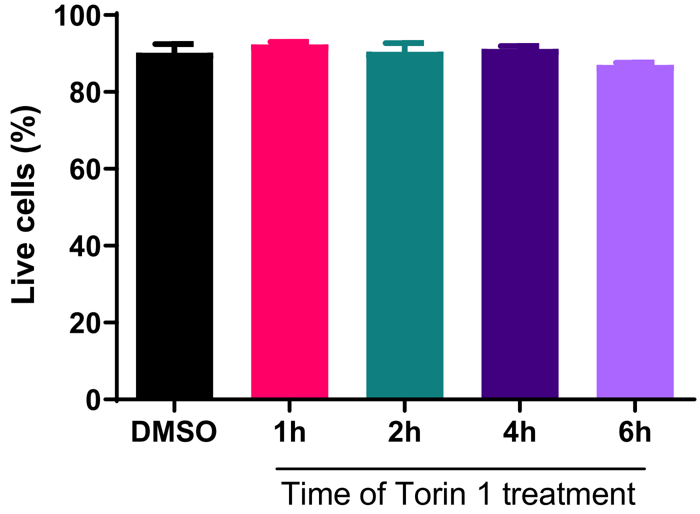

Figure S4. Torin 1 does not affect B cell viability. (A) Gating strategy of B cells treated with Torin 1 and stained for Annexin V and Propidium Iodide to measure apoptotic and necrotic cells by FACS. (B) Graph showing the percentage of live cells measured in the double negative quadrant for Annexin V and Propidium Iodide. N=3.

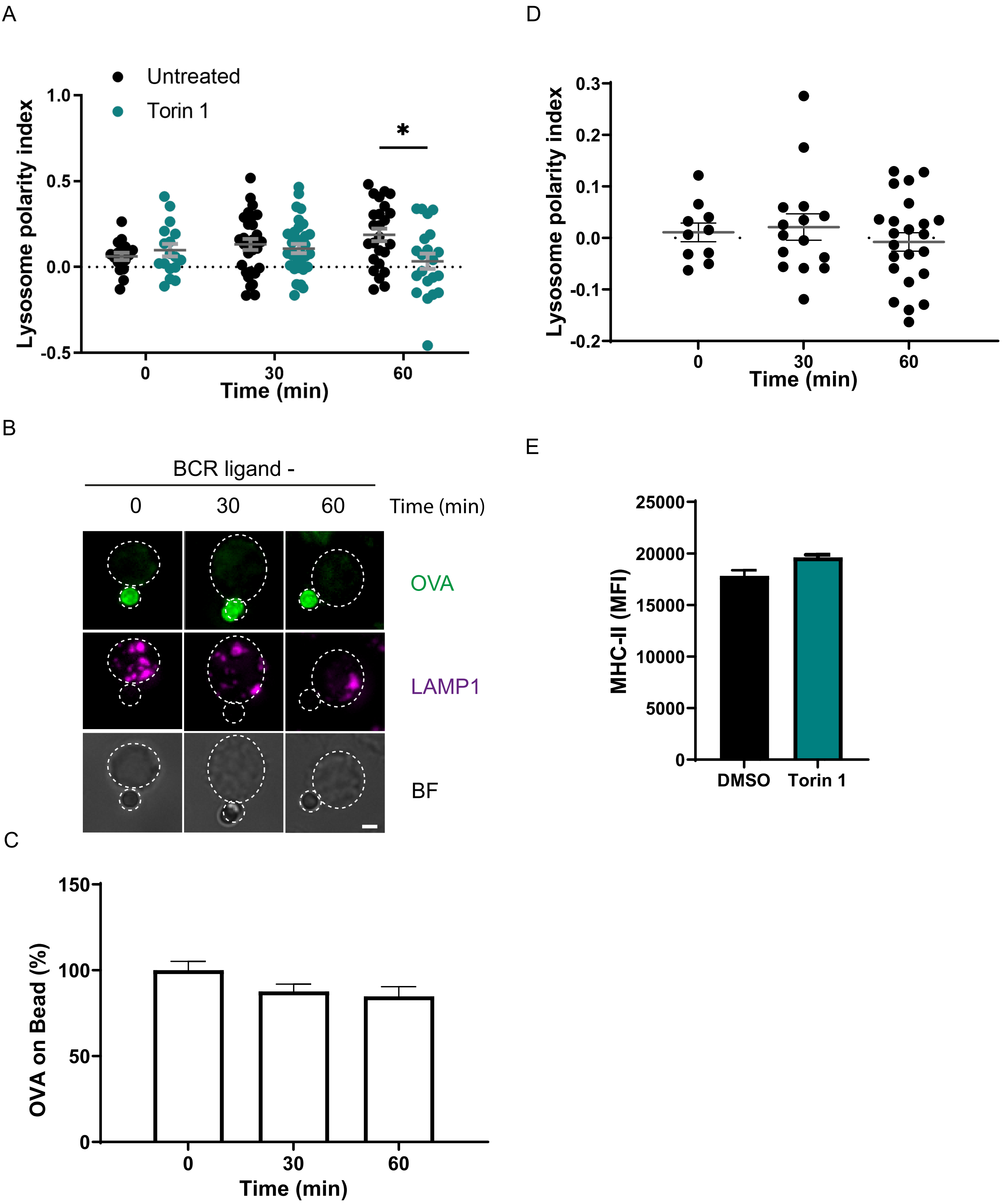

Figure S5. Torin 1 treated B cells fail to polarize lysosomes to the IS. (A) Lysosome polarity index from cells showed in figure 4B. *p<0.05. N=3. (>55 cells). Two-way ANOVA with Sidak's multiple comparison test. (B) Representative images Torin 1 treated B cells incubated with beads coated with OVA and a BCR ligand- (anti-IgM) in resting (0 min) and activated conditions. Cells were fixed and stained for OVA (green) and LAMP-1 (magenta). Images are shown as Z-projections of a stack. Scale bar = 3 µm. N=2 (B) Quantification of OVA fluorescence remaining on the bead, normalized by initial fluorescence of former cells. (D) Lysosome polarity indexes of cells of Figure S4 B. (E) Mean fluorescence intensity of MHC-II of untreated Torin 1 treated B cells (N=3).

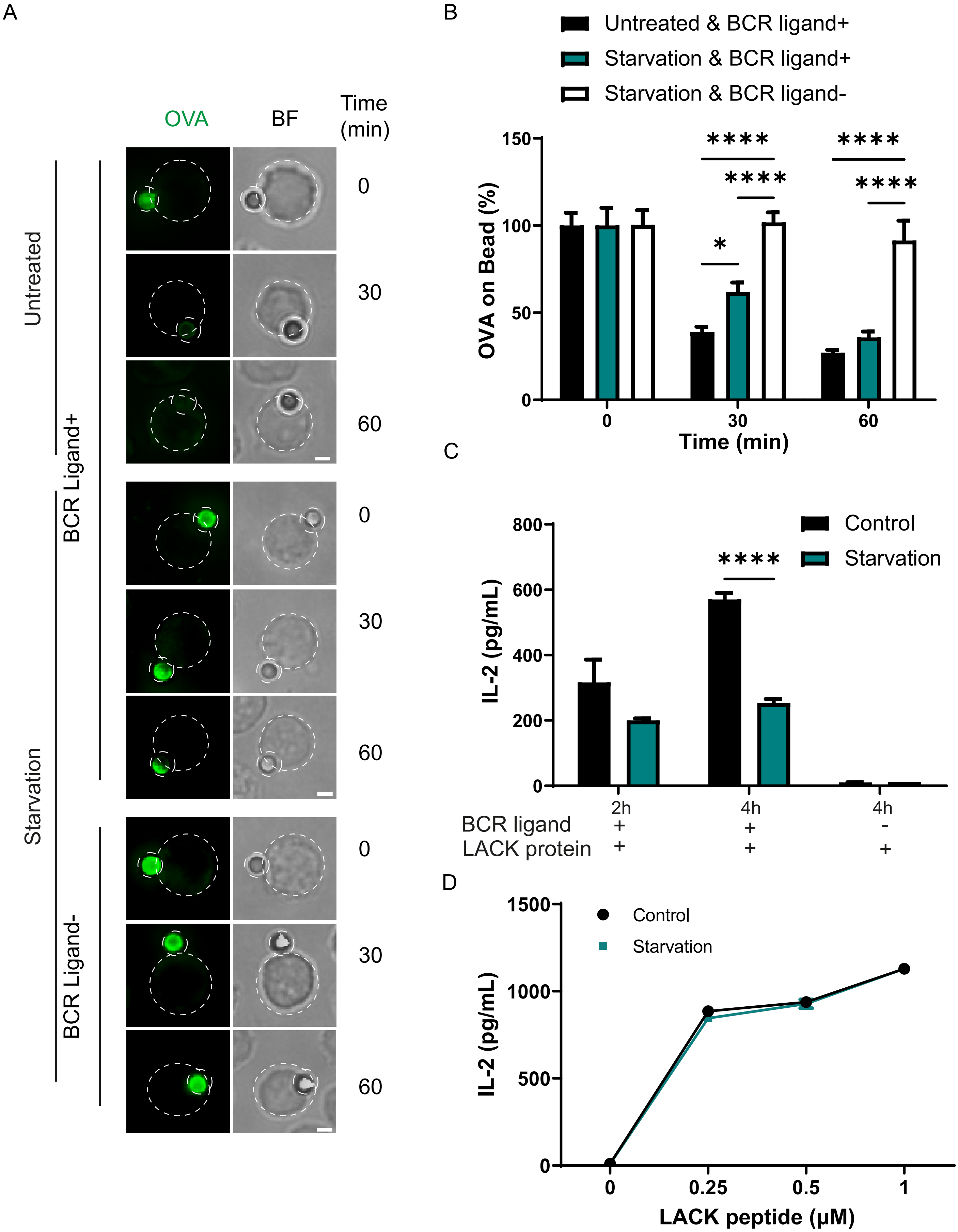

Figure S6. Induction of autophagy by starvation is sufficient to decrease antigen extraction and presentation in B cells. (A) Representative images of control and Torin 1 treated B cells incubated with beads coated with OVA and BCR ligand+ (anti-IgG) or BCR ligand- (IgM) for different time points. Cells were fixed and stained for OVA (green). Images are shown as Z-projections of a stack. Scale bar = 3 μm. N=3. BF: Bright Field (B) Quantification of OVA fluorescence remaining on the bead, normalized by initial fluorescence (time 0). ***p<0.001, ****p<0.0001. N=3. (>90 cells). Two-way ANOVA with Sidak's multiple comparison test. (C) Antigen presentation assay of control or starved B cells. Levels of IL-2 secretion by T cells were quantified by ELISA *p<0.05, ****p<0.0001. N=3. (D) Representative graph of peptide controls for cells used in antigen presentation assays.
